# Region-Dependent Alterations in Cognitive Function and ERK1/2 Signaling in the PFC in Rats after Social Defeat Stress

**DOI:** 10.1155/2018/9870985

**Published:** 2018-04-11

**Authors:** Qiong Wang, Feng Shao, Weiwen Wang

**Affiliations:** ^1^CAS Key Laboratory of Mental Health, Institute of Psychology, Beijing, China, School of Education, Zhengzhou University, Zhengzhou, China; ^2^The University of Chinese Academy of Sciences, Beijing, China; ^3^School of Psychological and Cognitive Sciences, Beijing Key Laboratory of Behavior and Mental Health, Peking University, Beijing, China

## Abstract

Cognitive dysfunctions are highly comorbid with depression. Impairments of cognitive flexibility, which are modulated by the monoaminergic system of the prefrontal cortex (PFC), are increasingly recognized as an important component of the pathophysiology and treatment of depression. However, the downstream molecular mechanisms remain unclear. Using a classical model of depression, this study investigated the effects of social defeat stress on emotional behaviors, on cognitive flexibility in the attentional set-shifting task (AST), and on the expression of extracellular signal-regulated kinase 1 and 2 (ERK1 and ERK2) and their downstream signaling molecules cAMP-response element binding protein (CREB) and brain-derived neurotrophic factor (BDNF) in two subregions of the PFC, the medial prefrontal cortex (mPFC), and the orbitofrontal cortex (OFC). The results showed that stress induced emotional and cognitive alterations associated with depression, including a decreased sucrose intake ratio and impaired reversal learning and set-shifting performance in the AST. Additionally, rats in the stress group showed a significant decrease only in ERK2 signaling in the mPFC, while more extensive decreases in both ERK1 signaling and ERK2 signaling were observed in the OFC. Along with the decreased ERK signaling, compared to controls, stressed rats showed downregulation of CREB phosphorylation and BDNF expression in both the OFC and the mPFC. Further analysis showed that behavioral changes were differentially correlated with several molecules in subregions of the PFC. These results suggested that social defeat stress was an effective animal model to induce both emotional and cognitive symptoms of depression and that the dysfunction of ERK signaling activities in the PFC might be a potential underlying biological mechanism.

## 1. Introduction

Depression is the most common serious psychiatric disorder among those with established sets of emotional and cognitive symptoms. Deficits in cognitive flexibility associated with prefrontal lobe dysfunction have been recognized as an important risk factor for the onset of depression [1, 2]. Additionally, antidepressant treatments, which ameliorate emotional symptoms but do not affect cognitive dysfunction, can predict the reoccurrence of depression and of worsened social function and adaptation [3, 4]. Studies across different species (human, primate, and rodent) have demonstrated that prefrontal monoaminergic systems, which are the main targets of antidepressants, are involved in the modulation of cognitive flexibility [5, 6]. For example, monoaminergic neurotransmitters, especially serotonin (5-HT) in the orbitofrontal cortex (OFC) and norepinephrine (NE) in the medial prefrontal cortex (mPFC), regulate reversal learning and set shifting, respectively, two core components of cognitive flexibility [7–10]. In addition, it has been shown that compared with acute antidepressant treatment, chronic antidepressant treatment has better outcomes for the amelioration of cognitive dysfunctions [11] and similar effects on emotional symptoms [12]. These data suggest that there may be a downstream molecular cascade involved in modulating the emotional and cognitive symptoms of depression.

The extracellular signal-regulated kinase (ERK) signaling pathway in neural cells is regarded as the aggregation point for the effects of monoamines in the brain [13]. Previous studies from us and others have shown that ERK signaling is widely involved in the regulation of neuronal plasticity [14], emotion [15], and learning and memory [16, 17]. For example, inhibition of mPFC ERK signaling pathways can induce depressive behaviors such as anhedonia [15] and impair the retention of fear memory [18]. Our previous study also found that acute microinjection of the stress hormone corticotropin-releasing hormone (CRH) into the locus coeruleus exerted an inverse U-shaped dose-response effect on the performance of cognitive flexibility, especially set shifting, and this effect was correlated with the level of ERK phosphorylation in the mPFC [19]. There are two isomers in the ERK family, ERK1 (42 kD) and ERK2 (44 kD). It has been shown that ERK1 and ERK2 have different roles in the regulation of cognitive function. For example, ERK2 mutant mice showed severe cognitive impairment in an associative learning task, and children with decreased ERK2 levels showed impaired cognitive function [20]. In contrast, there is an enhancement of striatum-dependent LTP in ERK1 knock-out mice, and the enhancement of ERK2 in ERK1 knock-out mice was strongly associated with an improvement in learning and memory [21]. However, it remains unclear whether and how ERK1 and 2 modulate different components of cognitive flexibility mediated by different subregions of the PFC.

The present study was designed to examine the effects of chronic stress on depressive-like behaviors and on the ERK1/2 pathways in different areas of the PFC, as well as on the relationships between those subregions. Social defeat stress, a classical animal model of depression [22], was used to induce depressive alterations with emotional and cognitive symptoms. Sucrose preference, a core parameter of depression, and cognitive flexibility were tested. Rodent cognitive flexibility was assessed via the attentional set-shifting task (AST), a task analogue to the Wisconsin card-sorting task, which sensitively reflects cortical function in humans. In addition, the activities of ERK1 and ERK2 signaling and their downstream targets cAMP-response element binding protein (CREB) and brain-derived neurotrophic factor (BDNF) in the mPFC and OFC were examined. These targets were chosen because CREB is one of the most important nuclear transcription factors downstream of ERK signaling [23], and it further regulates the production of BDNF, an activity-dependent molecule that exerts effects on neuronal activity [24]. There is also an activation of the ERK signaling pathway after binding of BDNF and its receptor TrkB. Therefore, the ERK pathway acts as both an upstream and a downstream signaling pathway of BDNF, both of which form a positive feedback loop [25–27].

## 2. Materials and Methods

### 2.1. Animals

Adult male Wistar rats (180–220 g) as intruders were obtained from the Lab Animal Center of the China Academy of Military Medical Sciences (Beijing, China). Animals arrived 7 days before the experiment for acclimation (days 1–7). Rats were maintained on a 12 h light/dark cycle (with the light on at 08:00 am) with food and water available ad libitum except for during the saccharine preference test and the restricted diet period in the AST. Body weights were determined once a week across the whole experimental period. As aggressive residents in the social defeat procedure, adult male Long-Evans rats (650–800 g) were obtained from the Lab Animal Center of the Third Military Medical University of the Chinese PLA (Chongqing, China) and singly housed for at least 14 days for a strong territorial effect.

All experimental procedures were performed with the approval of the Institutional Review Board of the Institute of Psychology at the Chinese Academy of Sciences and according to the guidelines of the National Institutes of Health Guide for the Care and Use of Laboratory Animals (NIH Publication number 80-23).

### 2.2. Social Defeat Stress

Chronic social defeat stress was widely used as an animal model of depression. After 7 days of acclimation, the intruder rats were randomly assigned to either the social defeat stress (*n* = 10) or the control (*n* = 6) groups. Rats in the stress group received 14 d of social defeat (days 8–21), and the controls were free of stress. The social defeat stress in this study was performed using the “resident-intruder” paradigm as described previously [28]. Briefly, each episode of social stress lasted for 45 min; during the first 15 min, a rat was placed into the home cage territory of an unfamiliar Long-Evans resident that was previously screened for high aggression, and the intruder faced different residents every day. A typical agonistic encounter resulted in intruder subordination or defeat, signaled by the intruder assuming a supine position for at least 3 sec. After the defeat, a wire mesh enclosure was placed in the cage to prevent physical contact between the resident and the intruder but allowing visual, auditory, and olfactory contact for the remaining 30 min of the defeat session. Controls were placed into a novel but unoccupied cage for 45 min daily in the same procedure. Rats were returned to their home cages after each session.

### 2.3. Sucrose Preference Test

Rats were deprived of water and food for 20 h (beginning at 20:00) and then were given a 2 h (16:00–18:00) time window for the sucrose preference test before (day 7) and after (day 21) 2 weeks of social defeat stress. The rats were given two bottles, one containing tap water and the other containing 1% sucrose solution. The amount of each solution consumed was determined by weighing the bottles before and after the test. Total sucrose solution intake and total water intake were recorded. The sucrose preference was assessed by calculating the percentage of sucrose solution intake as total sucrose solution intake/total (sucrose + water) intake. At the end of the preference test, rats were given free access to water and food.

### 2.4. AST

The procedures for the AST were similar to those described in our previous study [29]. Briefly, rats were restricted to 10–14 g of food per day to maintain 80–85% of their original body weight, with free access to water. Rats were trained to obtain a reward (1/4 of a Honey Nut Cheerio) by digging in two terracotta pots that were defined by a pair of cues along two stimulus dimensions: the digging medium filling the pots and the odor applied to the inner rim of the pots. The “positive” pot was baited with a reward buried at the bottom of the digging medium. The test contained five successive stages with increasing difficulty: the first stage was simple discrimination (SD), which only presented one relevant stimulus dimension (e.g., the medium). The second stage was compound discrimination (CD) in which the same relevant stimulus dimension as that in the SD stage (medium) was required, and the second dimension (e.g., the odor) was presented as an irrelevant distractor. The third stage was intradimensional shifting (IDS), wherein the medium was still the relevant dimension and the odor was still irrelevant, but new media and new odors were introduced. The fourth stage was reversal learning (REL), in which the same media and odors were used and the medium remained the relevant dimension, but the positive and negative cues from the IDS stage were reversed. The fifth stage was extradimensional shifting (EDS), in which all new media and odors were again introduced, and the relevant dimension was the odor instead of the medium. The test proceeded to the next stage when a rat reached a criterion of six consecutive correct trials. The number of trials to reach the criterion for each stage was recorded.

### 2.5. Tissue Sampling and Western Blotting Analysis

At 24 h after the end of the behavioral testing, rats were decapitated, and the brains were rapidly removed on ice. Each brain was placed into a freezing microtome (Leica, CM 3050, Germany), according to the atlas of Paxinos and Watson [30]; the mPFC (3.20–2.20 mm from the bregma) and OFC (4.70–3.70 mm from the bregma) were bilaterally punched using a stainless steel cannula with an inner diameter of 0.6 mm at −20°C as described in our previous studies [29, 31]. Then, the tissues were placed into liquid nitrogen for rapid freezing and were stored at −80°C for subsequent processing.

The tissue samples were placed in 50–70 *μ*L of precooled lysate buffer (4°C, pH 7.5, containing 5 *μ*g/mL leupeptin, 5 *μ*g/mL aprotinin, 5 *μ*g/mL pepsin inhibitor, 5 *μ*g/mL trypsin inhibitor, 2 mM EDTA, 2 mM EGTA, 1 mM DTT, and 0.5% NP-40) depending on their volume and were then homogenized using an ultrasonic homogenizer (Sonic Co., Stratford, CT, USA). The protein concentrations in the homogenates were determined by a bicinchoninic acid (BCA) Protein Assay Kit (CW Biotech, Beijing, China). The homogenates were then mixed with 5x sodium dodecyl sulfate (SDS) in proportion to the volume to prepare sample solutions with a certain concentration. The prepared sample solutions were denatured at 95°C for 8 min. Denatured proteins (32 *μ*g) were separated by 12% SDS-PAGE and transferred onto a nitrocellulose (NC) membrane at 230 mA for 1 h. The membrane was blocked with 5% nonfat milk diluted in TBST overnight at 4°C. After being washed in TBST (10 min × 3), the membrane was incubated at RT for 2 h on a shaker with primary antibodies: a rabbit monoclonal ERK1/2 antibody (1 : 1000, Cell Signaling Technology Inc., Beverly, MA, USA) and a rabbit monoclonal pERK1/2 antibody (1 : 2000, Cell Signaling Technology Inc.). After further washing in TBST (10 min × 3), the membrane was incubated at RT for 1 h on a shaker with an HRP-conjugated goat anti-rabbit IgG secondary antibody (1 : 4000, Zhongshan Golden Bridge Biotechnology, Beijing, China) and then washed again. Bands were detected by enhanced chemiluminescence (ECL, Millipore, Bedford, MA, USA) via a FluorChem E System (ProteinSimple, Santa Clara, CA, USA). After exposure, the membranes were stripped and reprobed with a primary mouse monoclonal GAPDH antibody (1 : 1000, Zhongshan Golden Bridge Biotechnology) and secondary HRP-conjugated goat anti-mouse IgG (1 : 4000, Zhongshan Golden Bridge Biotechnology) following the above steps. The pCREB and BDNF levels and the corresponding GAPDH levels were determined in different NC membranes using the same procedures. All bands were quantified using Lab Works TM 4.6 (image acquisition and analysis software). The ratio of the intensity of each target band to that of the GAPDH band was used to analyze differences between the stress and control groups.

### 2.6. Statistical Analysis

The statistical analysis was performed using “Statistical Package for Social Sciences” software (SPSS, version 11.5). AST data were analyzed by two-way ANOVA (stress × stage) with repeated measures over stages. The body weight and sucrose preference data were analyzed by two-way ANOVA (stress × test day) with repeated measures over tests. The levels of molecular expression in each subregion of the PFC between the stress and control groups were compared using Student's *t*-test. Pearson's correlation analysis was adopted for correlation analyses of molecular levels and behavioral alterations in the mPFC and OFC, respectively. Differences were considered significant at *p* < 0.05.

## 3. Results

### 3.1. Body Weight

**Figure 1:**
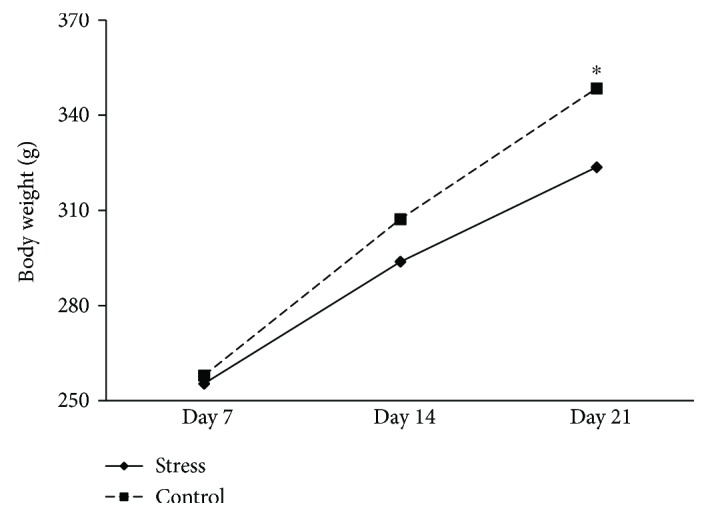
Body weights of controls and stressed rats before the stress (day 7), on the 7th day of stress (day 14), and on the 14th day of stress (day 21). Data are expressed as the mean ± SEM. ^∗^*p* < 0.05 compared to controls.

As shown in Figure 1, there were significant effects of the test day [*F*(2, 13) = 224.3, *p* < 0.001] and stress condition [*F*(1, 14) = 7.5, *p* = 0.016]. Further analysis showed that there was no difference in body weight between the controls and stressed animals (*t*_15_ = −0.713, *p* = 0.488) before stress exposure (day 7). Animals in the stress group showed a lower body weight compared to that in the controls after 7 days (*t*_15_ = −2.760, *p* = 0.015) and 14 days (*t*_15_ = −2.883, *p* = 0.0012) of stress exposure.

### 3.2. Sucrose Preference Test

**Figure 2:**
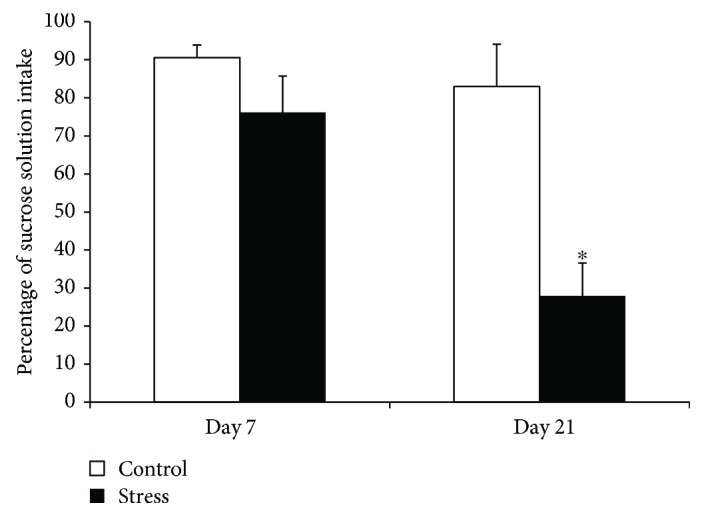
The percentage of sucrose solution intake in the control and stress groups before (day 7) and after stress (day 21). Data are expressed as the mean ± SEM. ^∗^*p* < 0.05 compared to controls.

The results indicated the main effects of the test day [*F*(1, 12) = 7.279, *p* = 0.019] and stress [*F*(1, 12) = 5.356, *p* = 0.039]. Further analysis showed that the percentage of sucrose solution intake was not significantly different (*t*_15_ = −0.309, *p* = 0.763) between controls and stressed animals before the stress; however, significant differences were observed after 14 days of stress exposure (*t*_15_ = −2.818, *p* = 0.014) (Figure 2).

### 3.3. AST

**Figure 3:**
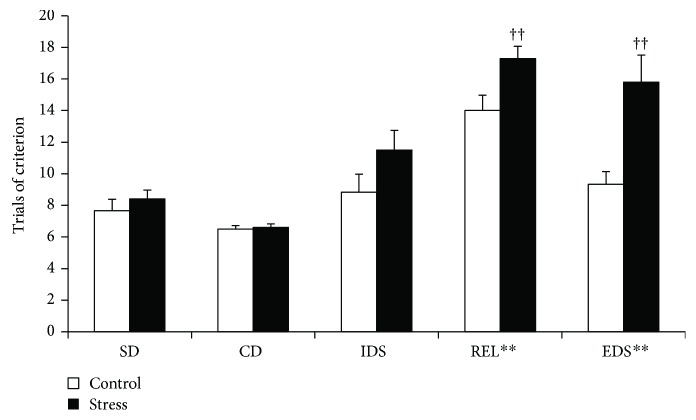
The performance in each stage of the AST in the control and stress groups after social defeat stress. ^++^*p* < 0.01. More trials to reach the criterion were required in the REL and EDS stages compared to those in the SD, CD, and IDS stages. Social defeat induced requirements for significantly higher numbers of trials to reach the criterion in the REL and EDS stages. ^∗∗^*p* < 0.01 compared to controls in the same stage.

Figure 3 shows significant main effects of stress [*F*(1, 14) = 23.35, *p* < 0.001] and task [*F*(4, 56) = 21.517, *p* < 0.001] and a significant effect of the stress × task interaction [*F*(4, 56) = 2.576, *p* = 0.047]. For the main effect of task, post hoc comparisons showed that significantly more trials were required to reach the criterion during REL and EDS than during the other tasks (*p* < 0.001). Post hoc analysis of the stress effect indicated that stressed rats required significantly more trials to reach the criterion in the REL and EDS stages of the AST than did the controls (REL: *t*_15_ = 2.672, *p* = 0.018; EDS: *t*_15_ = 3.41, *p* = 0.005; Figure 3).

### 3.4. Protein Expression of ERK1/2, CREB, and BDNF in the mPFC and OFC

**Figure 4:**
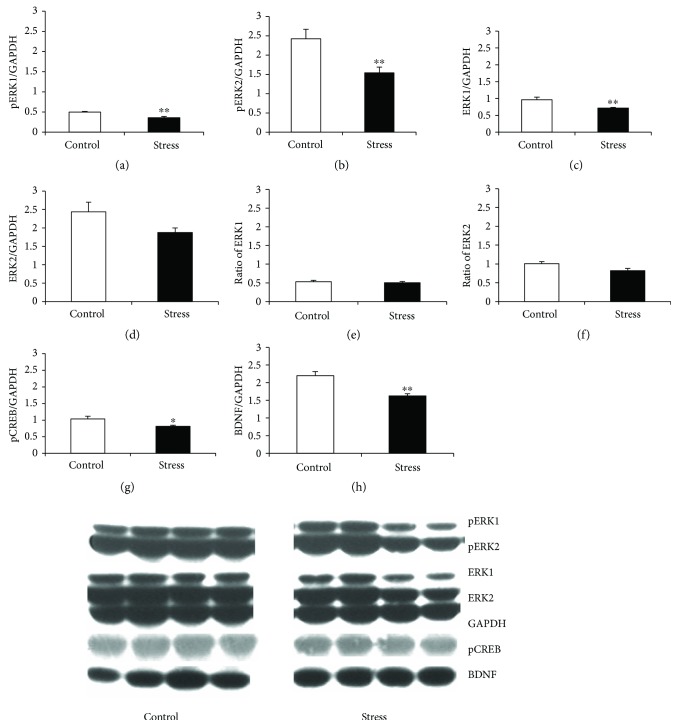
The levels of pERK1 and 2, ERK1 and 2, pCREB, and BDNF and representative blots for each protein from the OFC. (a) pERK1, (b) pERK2, (c) ERK1, (d) ERK2, (e) pERK1/ERK1, (f) pERK2/ERK2, (g) pCREB, and (h) BDNF. Data are expressed as the mean ± SEM. ^∗^*p* < 0.05 and ^∗∗^*p* < 0.01 compared to controls.

**Figure 5:**
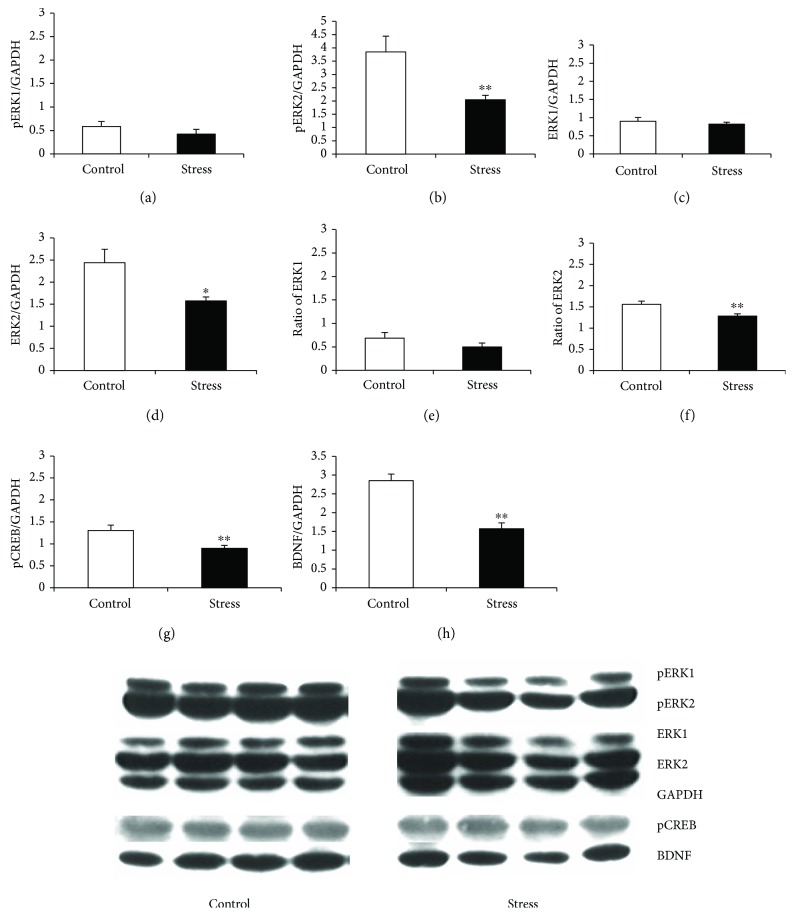
The levels of pERK1 and 2, ERK1 and 2, pCREB, and BDNF and representative blots for each protein from the mPFC. (a) pERK1, (b) pERK2, (c) ERK1, (d) ERK2, (e) pERK1/ERK1, (f) pERK2/ERK2, (g) pCREB, and (h) BDNF. Data are expressed as the mean ± SEM. ^∗^*p* < 0.05 and ^∗∗^*p* < 0.01 compared to controls.

The levels of pERK1/2, ERK1/2, pCREB, BDNF, and GAPDH in the OFC and mPFC are shown in Figures 4 and 5, respectively. Stress significantly decreased ERK1, pERK1, pERK2, pCREB, and BDNF levels in the OFC (ERK1: *t*_14_ = 3.774, *p* = 0.002; pERK1: *t*_14_ = 4.109, *p* = 0.001; pERK2: *t*_14_ = 3.27, *p* = 0.006; pCREB: *t*_14_ = 2.958, *p* = 0.011; and BDNF: *t*_14_ = 4.765, *p* < 0.001; Figure 4). There were no changes in ERK2, in the ratio of pERK1 to total ERK1 (pERK1/ERK1), or in the ratio of pERK2 to total ERK2 (pERK2/ERK2) in the OFC. Stress also markedly decreased pERK2, ERK2, pERK2/ERK2, pCREB, and BDNF levels in the mPFC (pERK2: *t*_14_ = 3.468, *p* = 0.004; ERK2: *t*_14_ = 2.74, *p* = 0.035; pERK2/ERK2: *t*_14_ = 13.00, *p* < 0.001; pCREB: *t*_14_ = 3.256, *p* = 0.006; and BDNF: *t*_14_ = 5.46, *p* < 0.001; Figure 5). There were no changes in pERK1, ERK1, or pERK1/ERK1 in the mPFC.

### 3.5. Correlations between Behavioral and Molecular Alterations in Rats

Results of the correlation analysis are shown in Table 1. In the mPFC, several molecules, including pERK2, ERK2, and BDNF, showed trends of negative association with the number of trials to reach the criterion in the EDS stage of the AST (*r* = −0.460, *p* = 0.085; *r* = −0.476, *p* = 0.073; and *r* = −0.485, *p* = 0.067, resp.), while a marginally or significantly positive correlation was observed between these proteins and sucrose preference (*r* = 0.473, *p* = 0.075; *r* = 0.482, *p* = 0.069; and *r* = 0.534, *p* = 0.040, resp.). In the OFC, we observed a significantly negative correlation only between the number of trials to reach the criterion in the REL stage and the BDNF expression level (*r* = −0.559, *p* = 0.030).

## 4. Discussion

In the present study, chronic social defeat stress induced behavioral and molecular alterations in depression, as manifested by anhedonia and deficits in cognitive flexibility, as well as by inhibited ERK-CREB-BDNF signaling in two subregions of the PFC. Further analysis showed a correlative relationship between behavioral and molecular alterations. These results suggested that the ERK signaling pathway may be a molecular mechanism for emotional and cognitive dysfunction in depression and is a potential target for better pharmaceutical treatment.

Social defeat stress induced a set of behavioral phenotypes of depression, including a decrease in the percentage of sucrose solution intake (reflecting anhedonia, a core symptom of depression) and reduced body weight gain, as well as increased trials to the criterion in the REL and EDS stages of the AST (reflecting deficits in cognitive flexibility in strategy shifting and set shifting, resp.). These results are in accord with those from other studies, in which repeated social defeat resulted in long-lasting loss of body weight [32, 33] and a decreased preference for sweet liquid in rats [34]. Social defeat stress did not significantly reduce sucrose preference of rats until the second week. Some researchers found that the effect of chronic stress on sucrose preference occurred around 10 days after stress [35, 36], while Becker et al. found that social defeat stress significantly decreased the sucrose preference within a short period of time [37]. In addition, other researchers reported that social defeat stress did not significantly change sucrose preference until the third week [38], which may be related to the different experimental paradigm and animal strains used in these studies. In addition, cognitive inflexibility is increasingly recognized as an important risk factor involved in the onset, treatment, and reoccurrence of depression [39]. The deficit profiles of various components of cognitive flexibility induced by chronic stress depend on their consequences on structure and function within the PFC. For example, chronic unpredictable stress and chronic restraint stress induced selective impairment in the EDS with no effect on the REL [11, 40], while chronic intermittent cold stress selectively impaired the REL in the AST but did not affect the EDS [41]. Using the social defeat stress model, this and previous studies by us and Snyder et al. reported impairment in cognitive flexibility in mice and rats [19, 29]. Considering social stress as the main source of life stress, these data suggested that chronic social defeat can be used as a validated social stressor to model both the emotional and cognitive symptoms of depression.

We found that ERK signaling in the PFC was inhibited by social defeat stress. There were significant decreases in the protein levels of ERK and/or phosphorylated ERK (pERK) in different subregions of the PFC, as shown by lower expression levels of ERK1, pERK1, and pERK2 in the OFC and of pERK2, ERK2, and pERK2/ERK2 in the mPFC in stressed rats compared to those in the control rats. Several studies have reported effects of various chronic stressors on ERK signaling in different brain areas. For example, chronic multiple stress impaired spatial cognition in the Morris water maze and significantly reduced the expression of pERK in the hippocampus and PFC of rats [42], while ERK activation caused the upregulation of dendritic spine density in CA1 pyramidal neurons [43–45] and improved spatial learning and memory [25, 46, 47]. It has been shown that social defeat stress can damage the structure and function of the PFC [48], which is involved in a variety of higher brain functions, such as emotion, social behavior, and cognitive function. Considering its important role in neuroplasticity, inhibition of ERK signaling in the mPFC and OFC in this study suggests that the deficits in cognitive flexibility may be mediated by impairment in corresponding areas.

In addition, this study showed differential alterations in ERK1 and ERK2 signaling in different areas of the PFC after social defeat stress, with a specific decrease in ERK2 signaling in the mPFC and a more extensive reduction in both ERK1 signaling and ERK2 signaling in the OFC. Functional differences in ERK1 and ERK2 signaling in the regulation of brain and behavior have been reported. For example, ERK2 plays a positive role in Ras-dependent cell proliferation, while ERK1 probably affects overall cell signaling output by antagonizing the activation of ERK2 [49]. Consistently, ERK1 knock-out mice exhibited increases in striatum-dependent LTP and ERK2 expression, and those effects were strongly associated [21]. A recent study also showed that ERK1 and ERK2 interacted to balance the process of cytoplasmic-nuclear trafficking [50]. Such differential effects of stress on ERK1 and 2 were also reported by Feng et al., in which depressed animals with early manipulation exhibited differential expression and phosphorylation of ERK1 and ERK2 in the hippocampus and in the frontal cortex [51], suggesting that a balance between ERK1 and 2 may be involved in neural and cognitive responses to stress.

Along with the inhibition of ERK signaling in the mPFC and OFC, expression levels of the downstream targets pCREB and BDNF were also significantly downregulated in this study. Various chronic stressors such as chronic social defeat can reduce CREB or pCREB and BDNF expression in the PFC [52–54], though studies do not show uniform results [55, 56] due to differences in experimental conditions, such as stress paradigms, brain areas analyzed, and time of testing. Decreasing or eliminating the expression of CREB or BDNF, due to stress or pharmaceutical or transgenic methods, causes emotional and cognitive impairment, such as in long-term memory consolidation [57] and in spatial cognition in the Morris water maze [33, 58]. In this report, we provide extensive evidence that ERK-CREB-BDNF signaling in the PFC may be involved in the alterations in cognitive flexibility induced by social defeat stress.

The relationship between ERK-CREB-BDNF signaling and the behaviors induced by social defeat were further supported by correlation analysis. First, the sucrose ratios were associated with BDNF levels in the mPFC. Other studies have shown that decreased BDNF expression in the hippocampus and mPFC was associated with chronic stress-induced depressive behaviors, such as anhedonia and despair behavior in the forced swimming test [59]. Snyder et al. found that there was a loss of sucrose preference in hippocampus neurogenesis-deficient mice than intact mice [60]. The present study also found that social defeat stress reduced BDNF expression in the mPFC and OFC, suggesting that the stress-induced reduction of sucrose preference may be related to changes of neurogenesis in the brain. Second, the increased number of trials to reach the criterion in the REL stage was significantly negatively correlated with the BDNF levels in the OFC. Structural and functional impairments in the OFC have been found in depressive patients and animals [61]. Considering that performance in reversal learning and set shifting depends on the normal structure and function of the serotoninergic system in the OFC and of the noradrenergic system in the mPFC, respectively [5, 8–10, 62, 63], these results suggest that different components of cognitive flexibility may be regulated by different ERK signaling pathways downstream of stress-induced changes in monoaminergic signaling, which shows extensive deficiencies in depression.

## 5. Conclusion

In conclusion, this study confirmed that social defeat stress can be used as a valid model to induce both the emotional and the cognitive domains of depression. Furthermore, we found that social defeat stress inhibited ERK-CREB-BDNF signaling by region-dependent effects on ERK1 and 2 in the PFC, which was correlated with anhedonia and impaired cognitive function. These results suggested that ERK-CREB-BDNF signaling in the PFC could be a common pathway involved in the emotional and cognitive symptoms of depression.

## Figures and Tables

**Table 1:** Correlations between emotional and cognitive alterations.

		Sucrose ratio	EDS
mPFC	pERK2	*r* = 0.473	*r* = −0.460
		*p* = 0.075	*p* = 0.085
	ERK2	*r* = 0.482	*r* = −0.476
		*p* = 0.069	*p* = 0.073
	pERK2/ERK2	*r* = 0.373	*r* = −0.380
		*p* = 0.171	*p* = 0.162
	pCREB	*r* = 0.355	*r* = −0.281
		*p* = 0.295	*p* = 0.310
	BDNF	*r* = 0.534^∗^	*r* = −0.485
		**p** = 0.040	*p* = 0.067
		Sucrose ratio	REL
OFC	pERK1	*r* = 0.362	*r* = −0.308
		*p* = 0.185	*p* = 0.263
	ERK1	*r* = 0.381	*r* = −0.306
		*p* = 0.162	*p* = 0.267
	pERK2	*r* = 0.409	*r* = −0.200
		*p* = 0.130	*p* = 0.476
	pERK2/ERK2	*r* = 0.360	*r* = −0.226
		*p* = 0.188	*p* = 0.419
	pCREB	*r* = 0.380	*r* = −0.127
		*p* = 0.162	*p* = 0.653
	BDNF	*r* = 0.333	*r* = −0.559^∗^
		*p* = 0.225	**p** = 0.030

^∗^ *p* < 0.05.

## Data Availability

The data used to support the findings of this study are available from the corresponding author upon request.
